# Stigma and public attitudes toward euthanasia or assisted suicide for psychiatric conditions: results from a general population survey in Germany

**DOI:** 10.1192/bjo.2024.4

**Published:** 2024-02-08

**Authors:** Georg Schomerus, Stephanie Schindler, Eva Baumann, Matthias C. Angermeyer

**Affiliations:** Department of Psychiatry, University of Leipzig Medical Center, Germany; Department of Journalism and Communication Research, Hannover University of Music, Drama, and Media, Germany; Center for Public Mental Health, Gösing am Wagram, Austria

**Keywords:** Human rights, stigma and discrimination, suicide, assisted suicide, euthanasia

## Abstract

**Background:**

With growing numbers of countries legalizing euthanasia or assisted suicide (EAS), there is a debate as to whether EAS should also be available to people with severe, treatment-resistant mental illness. Excluding mental illness as a legitimate reason to receive EAS has been framed as discriminating against people with mental illness.

**Aims:**

We examine whether approval or opposition to psychiatric EAS are related to stigma toward people with mental illness.

**Method:**

We asked a representative sample of the general population in Germany (*N* = 1515) whether they would approve of EAS for someone with severe, treatment-resistant mental illness. Stigma was assessed with the Value-Based Stigma Inventory (VASI), addressing rejection of people with mental illness in relation to different personal values.

**Results:**

A total of 19% of the German population approved of psychiatric EAS. Higher stigma scores were associated with greater approval of EAS (Spearman rank correlation coefficient, 0.28; *P* < 0.001). This association held true when controlling for sociodemographic variables. It was strongest for stigma related to perceived threats to one's security, reputation and meritocratic values.

**Conclusions:**

Our results highlight that, although opposing psychiatric EAS is sometimes framed as discriminatory, approval of psychiatric EAS might also carry hidden, stigmatising motives. To avoid any unintended negative consequences for people with severe, treatment-resistant mental illness, any legislation on psychiatric EAS needs to be crafted with particular caution.

A growing number of countries have legalised euthanasia or assisted suicide (EAS). Beyond EAS for severe physical disorders, there is a debate as to whether the right to receive medical assistance in dying should also extend to people with severe mental illness (psychiatric EAS). Few countries (Belgium, The Netherlands and Luxemburg) have included severe, treatment-resistant mental illness as a legitimate reason to request EAS.^1–4^ A recent systematic review by Nicolini and co-workers summarised arguments in favour of and against psychiatric EAS,^5^ showing that parity arguments figure most prominently in the debate: if EAS are permitted for severe physical disorders, it should also be permitted for severe psychiatric disorders. Denying EAS to people with mental disorders could be seen as a form of discrimination, falsely suggesting, for example, that ‘their suffering is more tolerable than that of people suffering from physical disorders’.^6^ On the other hand, the difficulties ‘to make objective the criterion of irremediability of a mental disorder; ( … ) to balance suicide prevention with assisted suicide; ( … ) to avoid the risk of progressively including in requests for [euthanasia or assisted suicide] vulnerable segments of the population, such as minors, elderly, or people with dementia, in a productive-oriented society’^7^ are reasons given against EAS for people with severe mental illness.

## Stigma, discrimination and EAS

Although existing laws on non-psychiatric EAS are the most obvious reference point for the psychiatric EAS debate, there is also a historical context: the history of involuntary euthanasia for people with severe chronic mental illness, culminating in a state-sponsored mass murder of more than 70 000 people in Nazi Germany.^8^ The systematic review by Nicolini et al did not report any arguments relating to this historical context. However, we believe that the history of involuntary euthanasia demands a particularly cautious debate on voluntary euthanasia. In particular, we need to be sensitive of any discriminatory motives in favour of psychiatric EAS, which might be hidden beneath a seemingly empowering narrative of patient autonomy and parity between mental and physical disorders. The current study aims to find out to what extent stigmatising attitudes are related to favourable attitudes toward voluntary psychiatric EAS.

Stigma has been conceptualised by Link and Phelan as a process of four interrelated components (labelling, stereotyping, separation, status loss and discrimination) in the context of a power gradient: a powerful group can use the stigma process to inflict status loss and discrimination on a group with less power.^9,10^ People with chronic severe mental disorder are a particularly vulnerable group, and thus are in particular danger for being harmed by stigma.

According to Yang et al's theory of ‘what matters most’,^11^ stigma is most visible in interactions or situations that matter most in a specific cultural context. A comparison between Tunisia and Germany, for example, showed that stigma was particularly visible in family relationships in Tunisia, whereas it was more pronounced in work-related situations in Germany.^12^ ‘What matters most’ has also been applied to personal values within a shared cultural context:^13^ members of minorities are more likely to face prejudice and discrimination if they are perceived as a threat to one's personal values.^11,14^ Hence, If someone is highly valuing security, they might reject someone with mental illness more strongly if they believe this person poses a danger to their security. Someone with strong meritocratic values, in turn, might reject someone with mental illness if they seem to enjoy benefits they do not deserve. So far, the stigma of mental illness has mostly been shown to be related to conservative or authoritarian values, centring around the stereotypes of dangerousness and unpredictability as threats to safety and order. Liberal values have been somewhat underresearched in this respect.^13^ Using a stigma scale that captures rejection in the context of a broad range of value orientations,^15^ our aim is to find out whether approval of psychiatric EAS is related to rejection of people with mental illness in general, and to what extent approval of psychiatric EAS is linked to rejection specifically for different value orientations.

## Method

### Sample

We conducted a face-to-face, paper-and-pencil survey in Germany in 2020 (*N* = 1515) among a representative probability sample of people living in private households, aged 18 years and older. The survey used a three-stage random sampling procedure with a random selection of (a) sample points (electoral wards), (b) households and (c) individuals within target households. Target households within sample points were determined according to the random route procedure; that is, a street was selected randomly as a starting point, from which interviewers followed a set route through the area. Target individuals within households were selected using random digits. Both target households and target individuals were approached up to four times if they were not available at the initial visit. Fieldwork was carried out by USUMA (Berlin), a company who specialise in market and social research. Table 1 shows the sociodemographic characteristics of our sample and of the general population in Germany in 2020. Compared with the general population, our sample contained slightly more women and people with a medium level of education (10 years of schooling). A total of 20.9% of our sample stated that they had received any kind of treatment or counselling for mental health issues in the past, and 14.5% reported this for someone within their family.

**Table 1:** Sociodemographic characteristics of the study sample and general population in Germany

	Survey, 2020 (%)	Total population, 2020 (%)
Gender		
Male	45.2	48.9
Female	54.5	51.1
Diverse	0.4	Not applicable
Age, years		
18–25	11.4	10.2
26–45	32.5	30.0
46–60	28.0	26.9
≥61	28.1	32.9
Educational attainment^a^		
Student/unknown	0.4	0.1
No schooling completed	1.5	4.1
8/9 years of schooling	28.9	28.5
10 years of schooling	42.6	32.0
12/13 years of schooling	26.6	35.2
Marital status		
Single	31.9	32.2
Married	46.0	50.7
Widowed	8.9	8.1
Divorced	13.2	9.1
Employment status^a^		
Employed	62.0	57.7
Unemployed	5.2	5.0
Retired	27.2	29.9
Other (e.g. apprenticeship)	5.6	7.4
Migration background^a^		
None	85.6	76.7
Self	8.0	18.5
Parents	6.4	4.9

Percentages of sample (*N* = 1515) and reference population aged 18 years and older if not otherwise specified. Population data from the Federal Statistical Office of Germany.^17^

a. For comparability, population and sample data (*n* = 1361) include only those aged ≥25 years.

The interview started with presenting respondents with a case vignette of someone with either schizophrenia or depression, without labelling the problem. The vignette was followed by questions relating to the problem described. In a second part of the interview, we asked questions unrelated to the case vignette. The present study is derived from data from this second, vignette-unrelated part. Although 3042 persons completed the interview for both vignettes (response rate of 57%),^16^ the questions relevant to this study were only asked in the random subsample of people receiving the depression vignette (*n* = 1530), and 1515 valid answers were obtained.

After having received written data protection information, participants gave verbal informed consent to participate in the interview, which was documented by the interviewer.

The authors assert that all procedures contributing to this work comply with the ethical standards of the relevant national and institutional committees on human experimentation and with the Helsinki Declaration of 1975, as revised in 2008. The study was approved by the review board of Greifswald University Medical Center (approval number BB 195/18).

### Agreement with psychiatric EAS

Participants used a five-point Likert-type scale from 1 (‘agree completely’) to 5 (‘disagree completely’) to indicate their agreement with the statement: ‘If there is no prospect of recovery, people with severe mental illness should have the opportunity to receive euthanasia or assisted suicide’. We used the German word ‘*sterbehilfe*’, which can be translated as both euthanasia and assisted suicide. Scores were inverted, so that higher values indicate greater agreement with psychiatric EAS.

### Stigma

We assessed stigma with the Value-Based Stigma Inventory (VASI).^15^ The VASI comprises 15 items assessing to what extent respondents perceive dealing with someone with mental illness as interfering with their personal priorities or values. In our sample, internal consistency (Cronbach's alpha) of the entire scale was 0.85. Five subscales of three items each can be distinguished, referring to different values (Table 2): security, reputation, meritocratic values, self-realization and personal growth. The VASI has been shown to be correlated with the desire for social distance (Social Distance Scale;^18,19^ *r* = 0.68) and with agreement with negative stereotypes about someone with mental illness (Self-Stigma of Mental Illness Scale,^20^ subscale ‘stereotype endorsement’; *r* = 0.67).^15^ All items were answered on five-point Likert-type scales from 1 (agree completely) to 5 (do not agree at all). Except for the items on personal growth, all scores were reversed, so that higher scores consistently reflect stronger stigmatising attitudes. Following the manual of the scale, to achieve the same value range for the total score and the five subscale sum scores, we divided the sum score of the entire scale by five, resulting in values ranging from 3 to 15 for all scores.

**Table 2:** Correlation of approval of psychiatric euthanasia or assisted suicide with stigma in a representative population sample in Germany

VASI (Cronbach's *α*)		Example item	Correlation with approval of psychiatric EAS	*n*	*P-*value
Full scale (0.85)			0.28	1475	<0.001***
Subscale	Security (0.81)	People with mental illness commit particularly cruel crimes	0.31	1495	<0.001***
	Reputation (0.82)	Having people with mental illness living in the neighbourhood reduces the attractiveness of my residential area	0.26	1503	<0.001***
	Meritocratic values (0.69)	Going easy on people with mental illness in the workplace is unfair to those who do not have a mental illness	0.23	1507	<0.001***
	Self-realisation (0.73)	If you are living together with a person with mental illness, it is difficult to lead a life according to your own ideas	0.11	1509	<0.001***
	Personal growth (0.74)	Interacting with people with mental illness can be very enriching for oneself	0.06	1500	0.015*

We report Spearman bivariate correlation coefficients. VASI subscale tests are corrected for multiple comparisons with the Bonferroni–Holm procedure. VASI, Value-Based Stigma Inventory; EAS, euthanasia or assisted suicide.

*P < 0.05, ***P < 0.001.

### Statistics

The distribution of the (inverted) values representing agreement with psychiatric EAS was left-skewed and platykurtic (skew: 0.630, kurtosis: –0.79), and indicated a marked preference for the extreme and neutral categories. Accordingly, we used nonparametric correlations (Spearman rank correlation coefficient, two-tailed tests) for a first, general impression of the relationships between psychiatric EAS and stigma as measured with the VASI (sum score and subscale scores), controlling the family-wise error rate for the five VASI subscale tests with the Bonferroni–Holm procedure. For the distributional characteristics of the study measures, please see Supplementary Table 1 available at https://doi.org/10.1192/bjo.2024.4.

In a second step, to achieve a more detailed understanding of the observed relationships and to control for potential confounders, we modelled support for psychiatric EAS as an ordinal outcome by using multinomial logistic regressions with the stigma measures (VASI total and subscales) as primary predictors. To secure high statistical power and to facilitate interpretation of our outcome measure, we collapsed the two responses below and above the middle category, creating the three levels ‘agree’, ‘neutral’ and ‘disagree’ with regard to psychiatric EAS. The disagreement category served as reference category for the estimated relative risk ratio of the neutral response and agreement. In addition, average marginal effects were calculated to describe the average change in response frequencies per unit increase in the VASI measure.

All multivariate models controlled for age, gender and education of the respondents. They also controlled for the interaction between education and VASI measure, because sensitivity analyses (not reported) of the bivariate correlation analyses had indicated that the relationship between stigma and psychiatric EAS might depend on educational attainment. To ensure stability of the model estimates, we excluded six subjects of diverse gender and collapsed educational attainment into three categories (<10, 10 and >10 years of schooling completed).

To assess sensitivity of our analyses for deviations from the reference population caused, for example, by systematic non-response, we repeated the multivariate analyses with probability weights matching the sample distribution to the population distribution, with respect to age, gender and region.

All analyses were done with Stata SE for Windows, version 16.0, except for (unbiased) sample estimates of skewness and kurtosis, which were calculated in line with the recommendations of Hodges et al,^21^ using the R package *psych* (version 2.2.9) by Revelle^22^ and R version 4.2.2 (R Core Team, R Foundation for Statistical Computing, Vienna, Austria; see https://www.R-project.org/).

## Results

When combining the two affirmative or rejecting answer categories of the five-point Likert-type item, 19% of the sample approved of psychiatric EAS, 23% were undecided and 58% were opposed. Respondents with higher stigma were more ready to endorse psychiatric EAS (Table 2: VASI sum score; Spearman rank correlation coefficient: 0.28, *P* < 0.001).

Looking at the subscales of our stigma measure, we saw the strongest relationships with increased approval of psychiatric EAS and perceived threat to one's security, reputation and meritocratic values (Table 2). Marginal, but also positive relationships were seen with a perceived impairment of self-realisation, whereas low expectations of personal growth were barely associated with support for psychiatric EAS.

**Fig. 1:**
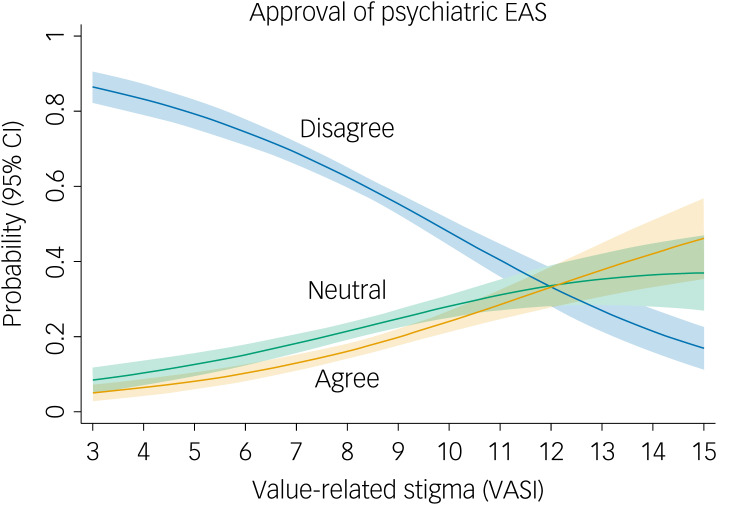
Predicted probabilities of approval of psychiatric euthanasia or assisted suicide (EAS) for different values of stigma (VASI sum score). Estimation sample size *N* = 1457. Multinomial regression analysis controlling for gender, education, age of respondents and the interaction between VASI and education. Shaded areas indicate 95% confidence intervals. VASI, Value-Based Stigma Inventory.

A detailed examination of the responses to our item on psychiatric EAS, using multinomial logistic regressions and controlling for age, gender, education and the interaction between education and the VASI, yielded positive significant relative risk ratios for the VASI total scale (relative risk ratios of 1.45 and 1.67 for neutral and positive attitudes; both *P* < 0.001), indicating that the proportion of both neutral and approving responses, relative to negative responses, increased with increasing stigma (Table 3). These estimates were almost exactly replicated in our sensitivity analysis using weighted survey data (Supplementary Table 2). Figure 1 depicts the predicted approval of EAS in relation to stigma (VASI sum score) in this multinomial regression model.

**Table 3:** Multinomial logistic regression analyses predicting approval of psychiatric euthanasia or assisted suicide

Predictor		*n*	Response category	Relative risk ratio [95% CI]	*Z*	*P-*value	Wald ‘s *χ*² (d.f. = 2)	*P-*value
VASI		1467	Disagree (reference)				53.08	<0.001***
			Neutral	1.45 [1.27–1.66]	5.46	<0.001***		
			Agree	1.68 [1.45–1.96]	6.72	<0.001***		
VASI subscale	Security	1487	Disagree (reference)				51.78	<0.001***
			Neutral	1.21 [1.13–1.31]	5.18	<0.001***		
			Agree	1.33 [1.22–1.45]	6.50	<0.001***		
	Reputation	1495	Disagree (reference)				39.43	<0.001***
			Neutral	1.18 [1.09–1.28]	3.98	<0.001***		
			Agree	1.31 [1.20–1.43]	5.97	<0.001***		
	Meritocratic values	1499	Disagree (reference)				57.71	<0.001***
			Neutral	1.29 [1.18–1.41]	5.48	<0.001***		
			Agree	1.45 [1.31–1.61]	7.07	<0.001***		
	Self-realisation	1501	Disagree (reference)				5.41	0.067
			Neutral	1.06 [0.98–1.16]	1.48	0.139		
			Agree	1.11 [1.01–1.22]	2.10	0.035*		
	Personal growth	1492	Disagree (reference)				6.10	0.047*
			Neutral	1.10 [1.00–1.20]	1.98	0.048*		
			Agree	1.11 [1.00–1.23]	1.91	0.056		

Selected results of the multinomial logistic regression analyses with euthanasia and assisted suicide as criterion, the VASI as primary predictor, and controlling for age, gender, educational attainment and the interaction of VASI×education. Response categories of euthanasia and assisted suicide were collapsed to represent agreement, neutral response and disagreement, with the latter serving as reference category. VASI, Value-Based Stigma Inventory.

*P < 0.05, ***P < 0.001.

Generally, although the predicted probability to oppose psychiatric EAS declined with higher stigma scores, both neutral and affirmative attitudes toward psychiatric EAS increased with rising stigma. Specifically, an overall increase of one point on the full VASI scale (median 8.2, range 3–15) would increase the proportion of affirmative answers on average across the full sample by 3.6%, increase the proportion of neutral responses by 3.0% and reduce the proportion of disagreement with EAS by 6.6%. This would amount to an increase in affirmative answers from 19 to 22%, an increase in neutral responses from 23 to 26% and a reduction in disagreement with psychiatric EAS from 58 to 51%.

For the subscales of our stigma measure, the regression models partly confirmed the bivariate correlations: we found the strongest effects with regard to stigmatising statements that were related to the security, reputation and meritocratic values of the respondents (Table 3).

Among the control variables, male gender was significantly (and reliably, in our sensitivity analyses) related to more neutral and approving responses regarding EAS. Higher education (>10 years) significantly and reliably predicted an overall increased agreement with psychiatric EAS in the models with the overall stigma score and the subscale of meritocratic values. Although higher age was generally associated with less agreement with psychiatric EAS, this relationship was below significance for most scales (Supplementary Tables 3–8). We also found indicators of an interaction between stigma and education: in respondents with higher educational achievement, the relationship between stigma and approval of EAS was weaker. Specifically, an increase in one point on the stigma scale (VASI sum score, range 3–15) would increase the proportion of affirmative answers among respondents with <10 years education by 4.6%, and by 2.5% among respondents with >10 years of education. For predictor estimates and model fit statistics of the multinomial regression analyses, see Supplementary Tables 3–8.

## Discussion

Our study shows that only a minority of people approve of EAS (‘*sterbehilfe*’) for people with mental illness in Germany, and this approval is associated with more stigma. This positive association between approving psychiatric EAS and rejecting people with mental illness in various contexts suggests that there may be other, hidden motives guiding our attitudes toward psychiatric EAS for people with severe mental illness beyond considerations for patient autonomy.

Before discussing our findings in detail, the limitations of our study need to be considered. First, we used a single-item measure of psychiatric EAS and were thus only able to capture general attitudes regarding this matter. Future studies could use case vignettes with different scenarios and diagnoses to elicit approval of EAS in specific situations, and could examine whether stigma has a differential impact on approval of EAS for different scenarios. We assume, for example, that priming with the schizophrenia vignette (instead of the depression vignette), although unrelated to our questions, could have resulted in an even stronger relationship between stigma and psychiatric EAS. Second, our stigma measure is unspecific with regard to the elements of the stigma process,^9^ containing items on negative stereotypes (‘Mental illness is often an excuse for laziness’), separation (‘In general, I feel comfortable spending time with a person with mental illness’, reversed) and discrimination (‘The neighbourhood should be warned about people with severe mental illness’). In fact, in a validation study using a large online sample, the VASI was correlated with both agreement with negative stereotypes and the desire for social distance.^15^ However, the VASI is uniquely specific with regard to values and preferences that may conflict with dealing with someone with mental illness, and thus provides us with new insights into potential motives behind stigmatising attitudes and their relationship to EAS, as we will discuss below. Third, we examined correlations and not causality. Experimental or quasi-experimental studies are needed to prove that changing stigma would also change attitudes toward psychiatric EAS. Fourth, our study was conducted in Germany, and in other countries the relationship between stigma and approval of psychiatric EAS might be different. Recent data from the European Values Study show that attitudes in Germany, at least with regard to euthanasia in general, are similar to attitudes in other Western European countries.^23^ Comparing attitudes toward psychiatric EAS and stigma in different countries with different legislation could prove valuable here, since it could also reveal whether the practice of psychiatric EAS affects the relationship between stigma and EAS approval. Finally, our study does not examine other potential motives behind the approval or rejection of psychiatric EAS. Approval of psychiatric EAS is certainly guided by more complex considerations that were not captured by our study.^5^ The aim of this paper is thus not to discredit arguments in favour of psychiatric EAS, but to add a note of caution.

The ‘what matters most’ theory by Yang and colleagues posits that stigma becomes most pervasive in situations that are particularly relevant in a specific cultural context.^11,13^ In focusing on personal values and preferences that may be perceived as being threatened when encountering someone with mental illness, our study highlights potentially competing motives that matter to respondents and, as our results suggest, make psychiatric EAS more permissible. Perceived threats to one's security, reputation and the meritocratic perception that people with mental illness are undeserving of the benefits they receive are all related to stronger approval of psychiatric EAS. Our results thus suggest that notions of dangerousness, being undeserving and fears concerning one's reputation should be addressed to reduce the influence of stigma on the discourse about psychiatric EAS.

Attitudes toward euthanasia in general have shifted in recent years. Data from the European Values Study show that in almost all countries in Europe, the population has become more favourable toward euthanasia over the past three decades. A particularly strong shift has occurred in Germany, which is now the country with the third most favourable opinion on this matter in Europe.^23^ This change is driven particularly by the opinion of younger people, but opinions changed toward more permissive attitudes within all age cohorts.^23^ The change in attitudes in Germany was particularly pronounced after the wording of the respective item in the European Values Study changed from ‘*euthanasie*’ to ‘*sterbehilfe*’ in 2008, the term we also used in this study. The term ‘*sterbehilfe*’ avoids the obvious historical ballast of ‘*euthanasie*’, which is closely associated with the non-voluntary killing of people with chronic mental illness and other disabilities during Nazi Germany.^24^ Today, Germany does not seem to be an outlier in Europe with regard to end-of-life attitudes.^23^

The European Values Study also shows that attitudes toward EAS are closely related to permissive attitudes about *in vitro* fertilisation, abortion, divorce and homosexuality,^24^ thus placing EAS within a context of other preferences for personal choices.^25^ Accordingly, when discussing arguments in favour of or against psychiatric EAS, stigma is usually referred to as an impediment to psychiatric EAS, diminishing the opportunities of people with mental illness to make personal choices. For example, in their systematic review of reasons for or against psychiatric EAS, Nicolini and co-workers mention that ‘Excluding the mentally ill [from EAS] based on “vulnerability” is discriminatory and stigmatizing’.^5^

Our data show, however, that attitudes toward psychiatric EAS are at least multifaceted. Alongside a ‘liberal’ motivation to enable and strengthen personal choices for people with severe mental illness with regard to EAS, there is another, hidden motive that seems contrary to these liberal tendencies; namely, the fact that people who reject people with severe mental illness are more likely to endorse psychiatric EAS. Since this relationship between stigma and EAS has not yet been discussed regarding EAS for people with severe mental illness, our results suggests that stigma is a hidden motive for endorsing psychiatric EAS that is not discussed openly.

Hidden motives, or undercurrents in public debate, are difficult to address or refute directly. Stigma that appears to be hidden beneath seemingly empowering narratives of personal choice is particularly difficult to address. Our study highlights the possibility that liberal legislation regarding psychiatric EAS, although seemingly contributing to parity between mental and medical disorders, could instead increase discrimination of people with severe mental illness and even become an element of structural stigma. As a practical conclusion to our study, we would thus argue that our finding, that approval of psychiatric EAS is positively related to stigmatising attitudes, at least demands a particularly critical and cautious approach to any potential legislation guiding psychiatric EAS, to avoid any unintended negative consequences for people with severe, treatment-resistant mental illness.

## Supporting information

Schomerus et al. supplementary material Schomerus et al. supplementary material

## Data Availability

The data that support the findings of this study are available on request from the corresponding author G.S., upon reasonable request.
