# Supplementary material for: Stigma and public attitudes toward euthanasia or assisted suicide for psychiatric conditions: results from a general population survey in Germany
Source: BJPsych Open. 2024 Feb 8;10(2):e44. doi: 10.1192/bjo.2024.4 (PMC10897688; doi:10.1192/bjo.2024.4)
Supplement: Schomerus et al. supplementary material [file S2056472424000048sup001.docx]

**Supplementary Material**

**Public attitudes toward euthanasia or assisted suicide for psychiatric conditions and stigma. Results from a general population survey in Germany.**

Georg Schomerus, Stephanie Schindler, Eva Baumann, Matthias C. Angermeyer

**Table S1**

**Summary statistics of the study measures.**

| Measure | | *N* | Central tendency | |  | Variability | | |  | Normality | |
| --- | --- | --- | --- | --- | --- | --- | --- | --- | --- | --- | --- |
|  |  |  | Mean | Median |  | *SD* | Range | *IQR* |  | Skew | Kurtosis |
| EAS | | 1515 | 2.29 | 2 |  | 1.32 | 1-5 | 2 |  | 0.63 | -0.79 |
| VASI | | 1475 | 8.43 | 8.2 |  | 1.98 | 3-15 | 2.6 |  | 0.44 | 0.34 |
|  | Security | 1495 | 7.80 | 8 |  | 3.34 | 3-15 | 5 |  | 0.37 | -0.70 |
|  | Reputation | 1503 | 5.88 | 5 |  | 2.89 | 3-15 | 5 |  | 0.90 | 0.05 |
|  | Meritocratic values | 1507 | 6.74 | 7 |  | 2.71 | 3-15 | 4 |  | 0.58 | -0.08 |
|  | Self-realization | 1509 | 10.67 | 11 |  | 2.64 | 3-15 | 4 |  | -0.36 | -0.17 |
|  | Personal growth | 1500 | 11.7 | 11 |  | 2.58 | 3-15 | 4 |  | -0.33 | -0.32 |

EAS euthanasia and assisted suicide (high values indicate strong endorsement), *N* sample size, *SD* standard deviation, *IQR* interquartile range, VASI Value-based Stigma Inventory (sum score and subscale scores; high values indicate strong endorsement of stigmatizing statements).

**Table S2**

**Sensitivity analysis of multinomial logistic regression results using weighted sample data.**

| Predictor | | *N* | Response category | Predictor estimates | | | | Predictor evaluation | | | Model evaluation | |
| --- | --- | --- | --- | --- | --- | --- | --- | --- | --- | --- | --- | --- |
|  |  |  |  | Relative risk ratio [95% CI] | *z* | *p* |  | Wald ‘s χ² (*df*=2) | *p* |  | Wald χ² (*df*=14) | Pseudo-*R*² (Mc-Fadden) |
| VASI | | 1467 | Disagree (Ref.) |  |  |  |  | 45.01 | <.001 | *** | 113.73*** | .051 |
|  |  |  | Neutral | 1.42 [1.24, 1.63] | 5.08 | <.001 | *** |  |  |  |  |  |
|  |  |  | Agree | 1.67 [1.42, 1.96] | 6.27 | <.001 | *** |  |  |  |  |  |
| VASI  Sub-scale | Security | 1487 | Disagree (Ref.) |  |  |  |  | 44.44 | <.001 | *** | 121.26*** | .050 |
|  |  |  | Neutral | 1.20 [1.12, 1.29] | 4.92 | <.001 | *** |  |  |  |  |  |
|  |  |  | Agree | 1.33 [1.21, 1.46] | 6.01 | <.001 | *** |  |  |  |  |  |
|  | Reputation | 1495 | Disagree (Ref.) |  |  |  |  | 30.96 | <.001 | *** | 88.75*** | .038 |
|  |  |  | Neutral | 1.16 [1.08, 1.26] | 3.78 | <.001 | *** |  |  |  |  |  |
|  |  |  | Agree | 1.31 [1.18, 1.44] | 5.28 | <.001 | *** |  |  |  |  |  |
|  | Meritocratic values | 1499 | Disagree (Ref.) |  |  |  |  | 54.21 | <.001 | *** | 90.47*** | .042 |
|  |  |  | Neutral | 1.31 [1.19, 1.45] | 5.55 | <.001 | *** |  |  |  |  |  |
|  |  |  | Agree | 1.46 [1.31, 1.63] | 6.92 | <.001 | *** |  |  |  |  |  |
|  | Self-realization | 1501 | Disagree (Ref.) |  |  |  |  | 6.20 | .045 | * | 44.82*** | .016 |
|  |  |  | Neutral | 1.05 [0.97, 1.14] | 1.14 | .254 |  |  |  |  |  |  |
|  |  |  | Agree | 1.13 [1.02, 1.24] | 2.43 | .015 | * |  |  |  |  |  |
|  | Personal growth | 1492 | Disagree (Ref.) |  |  |  |  | 4.18 | .124 |  | 27.87* | .011 |
|  |  |  | Neutral | 1.08 [0.98, 1.18] | 1.59 | .111 |  |  |  |  |  |  |
|  |  |  | Agree | 1.10 [0.98, 1.23] | 1.61 | .108 |  |  |  |  |  |  |

Selected results of the multinomial logistic regression analyses using probability weights that match the sample distribution to the population distribution with respect to age, gender, and federal state. CI confidence interval, *N* sample size, Ref. reference category, VASI Value-based Stigma Inventory.

* *p*<.05, *** *p*<.001.

**Table S3**

**Value-based stigma predicts EAS.**

| Predictor  (*N* = 1467) | Response category | Predictor estimates | | | Predictor evaluation | | | | Model evaluation | | | | |
| --- | --- | --- | --- | --- | --- | --- | --- | --- | --- | --- | --- | --- | --- |
|  |  | *b* (*SE*) | Relative risk ratio  [95% CI] | | Wald’s χ² (*df*=2) | | *p* | | | Likelihood-ratio χ² (*df*=14) | | Pseudo-*R*² (Mc-Fadden) | |
| VASI | Disagree (Ref.) |  |  |  | 53.08 | <.001*** | | 148.66*** | | | .053 | |  |
|  | Neutral | 0.372 (0.068) | 1.451 | [1.270, 1.659] |  |  |  |  |  |  |  |  |  |
|  | Agree | 0.520 (0.077) | 1.682 | [1.445, 1.957] |  |  |  |  |  |  |  |  |  |
| Age (years) | Disagree (Ref.) |  |  |  | 5.20 | .074 | |  |  |  |  |  |  |
|  | Neutral | -0.008 (0.004) | 0.992 | [0.984, 1.000] |  |  |  |  |  |  |  |  |  |
|  | Agree | -0.007 (0.004) | 0.993 | [0.985, 1.002] |  |  |  |  |  |  |  |  |  |
| Gender (0/1: female/male) | Disagree (Ref.) |  |  |  | 7.57 | .023* | |  |  |  |  |  |  |
|  | Neutral | 0.266 (0.134) | 1.305 | [1.004, 1.697] |  |  |  |  |  |  |  |  |  |
|  | Agree | 0.350 (0.145) | 1.420 | [1.068, 1.888] |  |  |  |  |  |  |  |  |  |
| Education (0/1: <10 years/ 10 years) | Disagree (Ref.) |  |  |  | 8.79 | .012* | |  |  |  |  |  |  |
|  | Neutral | 1.808 (0.771) | 6.098 | [1.346, 27.631] |  |  |  |  |  |  |  |  |  |
|  | Agree | 2.261 (0.900) | 9.588 | [1.643, 55.940] |  |  |  |  |  |  |  |  |  |
| Education (0/2: <10 years/ >10 years) | Disagree (Ref.) |  |  |  | 10.38 | .006** | |  |  |  |  |  |  |
|  | Neutral | 0.756 (0.845) | 2.129 | [0.406,11.166] |  |  |  |  |  |  |  |  |  |
|  | Agree | 2.999 (0.934) | 20.059 | [3.217, 125.068] |  |  |  |  |  |  |  |  |  |
| VASI*Education (0/1: <10 years/ 10 years) | Disagree (Ref.) |  |  |  | 7.35 | .025* | |  |  |  |  |  |  |
|  | Neutral | -0.194 (0.086) | 0.824 | [0.696, 0.975] |  |  |  |  |  |  |  |  |  |
|  | Agree | -0.220 (0.096) | 0.803 | [0.665, 0.969] |  |  |  |  |  |  |  |  |  |
| VASI*Education (0/2: <10 years/ >10 years) | Disagree (Ref.) |  |  |  | 7.23 | .027* | |  |  |  |  |  |  |
|  | Neutral | -0.066 (0.096) | 0.936 | [0.776, 1.130] |  |  |  |  |  |  |  |  |  |
|  | Agree | -0.273 (0.103) | 0.761 | [0.622, 0.931] |  |  |  |  |  |  |  |  |  |

Results of the multinomial logistic regression analyses with the VASI scale, age, gender, educational attainment, and the interaction of VASI*education as predictors of psychiatric EAS. Response categories of EAS were collapsed to represent agreement, neutral response, and disagreement with the latter serving as reference category. CI confidence interval, *df* degrees of freedom, EAS euthanasia and assisted suicide, *N* sample size, Ref. reference category, *SE* standard error, VASI Value-based Stigma Inventory.

* *p*<.05, ** *p*<.01, *** *p*<.001.

**Table S4**

**Stigma related to security concerns predicts EAS.**

| Predictor  (*N* = 1487) | Response category | Predictor estimates | | | Predictor evaluation | | | | Model evaluation | | | | |
| --- | --- | --- | --- | --- | --- | --- | --- | --- | --- | --- | --- | --- | --- |
|  |  | *b* (*SE*) | Relative risk ratio  [95% CI] | | Wald’s χ² (*df*=2) | | *p* | | | Likelihood-ratio χ² (*df*=14) | | Pseudo-*R*² (Mc-Fadden) | |
| VASI subscale Security | Disagree (Ref.) |  |  |  | 51.78 | <.001*** | | 146.58*** | | | .051 | |  |
|  | Neutral | 0.193 (0.037) | 1.213 | [1.128, 1.305] |  |  |  |  |  |  |  |  |  |
|  | Agree | 0.284 (0.044) | 1.329 | [1.220, 1.448] |  |  |  |  |  |  |  |  |  |
| Age (years) | Disagree (Ref.) |  |  |  | 3.90 | .142 | |  |  |  |  |  |  |
|  | Neutral | -0.007 (0.004) | 0.993 | [0.985, 1.000] |  |  |  |  |  |  |  |  |  |
|  | Agree | -0.005 (0.004) | 0.995 | [0.987, 1.003] |  |  |  |  |  |  |  |  |  |
| Gender (0/1: female/male) | Disagree (Ref.) |  |  |  | 9.87 | .007** | |  |  |  |  |  |  |
|  | Neutral | 0.311 (0.132) | 1.365 | [1.055, 1.767] |  |  |  |  |  |  |  |  |  |
|  | Agree | 0.387 (0.144) | 1.473 | [1.110, 1.954] |  |  |  |  |  |  |  |  |  |
| Education (0/1: <10 years/ 10 years) | Disagree (Ref.) |  |  |  | 6.88 | .032* | |  |  |  |  |  |  |
|  | Neutral | 0.937 (0.422) | 2.552 | [1.116, 5.834] |  |  |  |  |  |  |  |  |  |
|  | Agree | 1.008 (0.530) | 2.739 | [0.969, 7.741] |  |  |  |  |  |  |  |  |  |
| Education (0/2: <10 years/ >10 years) | Disagree (Ref.) |  |  |  | 7.28 | .026* | |  |  |  |  |  |  |
|  | Neutral | 0.292 (0.473) | 1.339 | [0.530, 3.384] |  |  |  |  |  |  |  |  |  |
|  | Agree | 1.478 (0.548) | 4.386 | [1.498, 12.843] |  |  |  |  |  |  |  |  |  |
| VASI*Education (0/1: <10 years/ 10 years) | Disagree (Ref.) |  |  |  | 6.25 | .044* | |  |  |  |  |  |  |
|  | Neutral | -0.110 (0.048) | 0.896 | [0.816, 0.984] |  |  |  |  |  |  |  |  |  |
|  | Agree | -0.093 (0.055) | 0.912 | [0.819, 1.015] |  |  |  |  |  |  |  |  |  |
| VASI*Education (0/2: <10 years/ >10 years) | Disagree (Ref.) |  |  |  | 3.89 | .143 | |  |  |  |  |  |  |
|  | Neutral | -0.028 (0.055) | 0.973 | [0.874, 1.083] |  |  |  |  |  |  |  |  |  |
|  | Agree | -0.117 (0.060) | 0.889 | [0.791, 1.000] |  |  |  |  |  |  |  |  |  |

Results of the multinomial logistic regression analyses with the VASI subscale Security, age, gender, educational attainment, and the interaction of VASI*education as predictors of psychiatric EAS. Response categories of EAS were collapsed to represent agreement, neutral response, and disagreement with the latter serving as reference category. CI confidence interval, *df* degrees of freedom, EAS euthanasia and assisted suicide, *N* sample size, Ref. reference category, *SE* standard error, VASI Value-based Stigma Inventory.

* *p*<.05, ** *p*<.01, *** *p*<.001.

**Table S5**

**Stigma related to perceived threats to one's reputation predicts EAS.**

| Predictor  (*N* = 1495) | Response category | Predictor estimates | | | Predictor evaluation | | | | Model evaluation | | | | |
| --- | --- | --- | --- | --- | --- | --- | --- | --- | --- | --- | --- | --- | --- |
|  |  | *b* (*SE*) | Relative risk ratio  [95% CI] | | Wald’s χ² (*df*=2) | | *p* | | | Likelihood-ratio χ² (*df*=14) | | Pseudo-*R*² (Mc-Fadden) | |
| VASI subscale Reputation | Disagree (Ref.) |  |  |  | 39.43 | <.001*** | | 113.54*** | | | .039 | |  |
|  | Neutral | 0.163 (0.041) | 1.177 | [1.086, 1.275] |  |  |  |  |  |  |  |  |  |
|  | Agree | 0.272 (0.046) | 1.312 | [1.200, 1.434] |  |  |  |  |  |  |  |  |  |
| Age (years) | Disagree (Ref.) |  |  |  | 2.73 | .255 | |  |  |  |  |  |  |
|  | Neutral | -0.006 (0.004) | 0.994 | [0.987, 1.002] |  |  |  |  |  |  |  |  |  |
|  | Agree | -0.005 (0.004) | 0.996 | [0.987, 1.004] |  |  |  |  |  |  |  |  |  |
| Gender (0/1: female/male) | Disagree (Ref.) |  |  |  | 9.06 | .011* | |  |  |  |  |  |  |
|  | Neutral | 0.285 (0.131) | 1.330 | [1.029, 1.720] |  |  |  |  |  |  |  |  |  |
|  | Agree | 0.374 (0.142) | 1.453 | [1.100, 1.919] |  |  |  |  |  |  |  |  |  |
| Education (0/1: <10 years/ 10 years) | Disagree (Ref.) |  |  |  | 8.07 | .018* | |  |  |  |  |  |  |
|  | Neutral | 0.513 (0.367) | 1.671 | [0.814, 3.428] |  |  |  |  |  |  |  |  |  |
|  | Agree | 1.194 (0.434) | 3.301 | [1.410, 7.732] |  |  |  |  |  |  |  |  |  |
| Education (0/2: <10 years/ >10 years) | Disagree (Ref.) |  |  |  | 5.06 | .080 | |  |  |  |  |  |  |
|  | Neutral | -0.086 (0.412) | 0.918 | [0.409, 2.058] |  |  |  |  |  |  |  |  |  |
|  | Agree | 0.974 (0.468) | 2.650 | [1.058, 6.632] |  |  |  |  |  |  |  |  |  |
| VASI*Education (0/1: <10 years/ 10 years) | Disagree (Ref.) |  |  |  | 5.83 | .054 | |  |  |  |  |  |  |
|  | Neutral | -0.067 (0.053) | 0.935 | [0.843, 1.038] |  |  |  |  |  |  |  |  |  |
|  | Agree | -0.138 (0.058) | 0.872 | [0.778, 0.976] |  |  |  |  |  |  |  |  |  |
| VASI*Education (0/2: <10 years/ >10 years) | Disagree (Ref.) |  |  |  | 2.68 | .262 | |  |  |  |  |  |  |
|  | Neutral | 0.049 (0.062) | 1.050 | [0.930, 1.185] |  |  |  |  |  |  |  |  |  |
|  | Agree | -0.064 (0.066) | 0.938 | [0.824, 1.067] |  |  |  |  |  |  |  |  |  |

Results of the multinomial logistic regression analyses with the VASI subscale Reputation, age, gender, educational attainment, and the interaction of VASI*education as predictors of psychiatric EAS. Response categories of EAS were collapsed to represent agreement, neutral response, and disagreement with the latter serving as reference category. CI confidence interval, *df* degrees of freedom, EAS euthanasia and assisted suicide, *N* sample size, Ref. reference category, *SE* standard error, VASI Value-based Stigma Inventory.

* *p*<.05, *** *p*<.001.

**Table S6**

**Stigma related to perceived threats to one's meritocratic values predicts EAS.**

| Predictor  (*N* = 1499) | Response category | Predictor estimates | | | Predictor evaluation | | | | Model evaluation | | | | |
| --- | --- | --- | --- | --- | --- | --- | --- | --- | --- | --- | --- | --- | --- |
|  |  | *b* (*SE*) | Relative risk ratio  [95% CI] | | Wald’s χ² (*df*=2) | | *p* | | | Likelihood-ratio χ² (*df*=14) | | Pseudo-*R*² (Mc-Fadden) | |
| VASI subscale Meritocratic values | Disagree (Ref.) |  |  |  | 57.71 | <.001*** | | 116.86*** | | | .040 | |  |
|  | Neutral | 0.252 (0.046) | 1.287 | [1.176, 1.408] |  |  |  |  |  |  |  |  |  |
|  | Agree | 0.371 (0.053) | 1. 449 | [1.308, 1.606] |  |  |  |  |  |  |  |  |  |
| Age (years) | Disagree (Ref.) |  |  |  | 5.00 | .082 | |  |  |  |  |  |  |
|  | Neutral | -0.008 (0.004) | 0.992 | [0.985, 1.000] |  |  |  |  |  |  |  |  |  |
|  | Agree | -0.007 (0.004) | 0.993 | [0.985, 1.001] |  |  |  |  |  |  |  |  |  |
| Gender (0/1: female/male) | Disagree (Ref.) |  |  |  | 10.79 | .005** | |  |  |  |  |  |  |
|  | Neutral | 0.301 (0.131) | 1.352 | [1.045, 1.748] |  |  |  |  |  |  |  |  |  |
|  | Agree | 0.415 (0.142) | 1.515 | [1.147, 2.001] |  |  |  |  |  |  |  |  |  |
| Education (0/1: <10 years/ 10 years) | Disagree (Ref.) |  |  |  | 20.43 | <.001*** | |  |  |  |  |  |  |
|  | Neutral | 1.248 (0.455) | 3.482 | [1.429, 8.487] |  |  |  |  |  |  |  |  |  |
|  | Agree | 2.282 (0.539) | 9.797 | [3.407, 28.170] |  |  |  |  |  |  |  |  |  |
| Education (0/2: <10 years/ >10 years) | Disagree (Ref.) |  |  |  | 21.23 | <.001*** | |  |  |  |  |  |  |
|  | Neutral | 0.702 (0.488) | 2.017 | [0.774, 5.253] |  |  |  |  |  |  |  |  |  |
|  | Agree | 2.556 (0.555) | 12.890 | [4.345, 38.241] |  |  |  |  |  |  |  |  |  |
| VASI*Education (0/1: <10 years/ 10 years) | Disagree (Ref.) |  |  |  | 19.31 | <.001*** | |  |  |  |  |  |  |
|  | Neutral | -0.168 (0.059) | 0.846 | [0.753, 0.950] |  |  |  |  |  |  |  |  |  |
|  | Agree | -0.276 (0.066) | 0.759 | [0.667, 0.863] |  |  |  |  |  |  |  |  |  |
| VASI*Education (0/2: <10 years/ >10 years) | Disagree (Ref.) |  |  |  | 16.50 | <.001*** | |  |  |  |  |  |  |
|  | Neutral | -0.077 (0.065) | 0.926 | [0.815, 1.053] |  |  |  |  |  |  |  |  |  |
|  | Agree | -0.291 (0.072) | 0.748 | [0.650, 0.861] |  |  |  |  |  |  |  |  |  |

Results of the multinomial logistic regression analyses with the VASI subscale Meritocratic values, age, gender, educational attainment, and the interaction of VASI*education as predictors of psychiatric EAS. Response categories of EAS were collapsed to represent agreement, neutral response, and disagreement with the latter serving as reference category. CI confidence interval, *df* degrees of freedom, EAS euthanasia and assisted suicide, *N* sample size, Ref. reference category, *SE* standard error, VASI Value-based Stigma Inventory.

** *p*<.01, *** *p*<.001.

**Table S7**

**Stigma related to perceived threats to one's self-realization predicts EAS.**

| Predictor  (*N* = 1501) | Response category | Predictor estimates | | | Predictor evaluation | | | | Model evaluation | | | | |
| --- | --- | --- | --- | --- | --- | --- | --- | --- | --- | --- | --- | --- | --- |
|  |  | *b* (*SE*) | Relative risk ratio  [95% CI] | | Wald’s χ² (*df*=2) | | *p* | | | Likelihood-ratio χ² (*df*=14) | | Pseudo-*R*² (Mc-Fadden) | |
| VASI subscale Self-realization | Disagree (Ref.) |  |  |  | 5.408 | .067 | | 51.36*** | | | .018 | |  |
|  | Neutral | 0.063 (0.043) | 1.065 | [0.980, 1.157] |  |  |  |  |  |  |  |  |  |
|  | Agree | 0.103 (0.049) | 1.109 | [1.007, 1.221] |  |  |  |  |  |  |  |  |  |
| Age (years) | Disagree (Ref.) |  |  |  | 6.013 | .049* | |  |  |  |  |  |  |
|  | Neutral | -0.008 (0.004) | 0.992 | [0.985, 0.999] |  |  |  |  |  |  |  |  |  |
|  | Agree | -0.008 (0.004) | 0.993 | [0.985, 1.001] |  |  |  |  |  |  |  |  |  |
| Gender (0/1: female/male) | Disagree (Ref.) |  |  |  | 14.818 | <.001*** | |  |  |  |  |  |  |
|  | Neutral | 0.346 (0.128) | 1.414 | [1.099, 1.818] |  |  |  |  |  |  |  |  |  |
|  | Agree | 0.473 (0.139) | 1.605 | [1.221, 2.109] |  |  |  |  |  |  |  |  |  |
| Education (0/1: <10 years/ 10 years) | Disagree (Ref.) |  |  |  | 0.216 | .897 | |  |  |  |  |  |  |
|  | Neutral | -0.127 (0.640) | 0.881 | [0.251, 3.087] |  |  |  |  |  |  |  |  |  |
|  | Agree | -0.343 (0.753) | 0.710 | [0.162, 3.105] |  |  |  |  |  |  |  |  |  |
| Education (0/2: <10 years/ >10 years) | Disagree (Ref.) |  |  |  | 0.004 | .998 | |  |  |  |  |  |  |
|  | Neutral | -0.039 (0.698) | 0.962 | [0.245, 3.777] |  |  |  |  |  |  |  |  |  |
|  | Agree | 0.011 (0.792) | 1.011 | [0.214, 4.771] |  |  |  |  |  |  |  |  |  |
| VASI*Education (0/1: <10 years/ 10 years) | Disagree (Ref.) |  |  |  | 0.563 | .755 | |  |  |  |  |  |  |
|  | Neutral | 0.017 (0.058) | 1.017 | [0.909, 1.139] |  |  |  |  |  |  |  |  |  |
|  | Agree | 0.049 (0.066) | 1.050 | [0.923, 1.195] |  |  |  |  |  |  |  |  |  |
| VASI*Education (0/2: <10 years/ >10 years) | Disagree (Ref.) |  |  |  | 0.158 | .924 | |  |  |  |  |  |  |
|  | Neutral | 0.009 (0.063) | 1.009 | [0.892, 1.142] |  |  |  |  |  |  |  |  |  |
|  | Agree | 0.028 (0.070) | 1.028 | [0.896, 1.179] |  |  |  |  |  |  |  |  |  |

Results of the multinomial logistic regression analyses with the VASI subscale Self-realization, age, gender, educational attainment, and the interaction of VASI*education as predictors of psychiatric EAS. Response categories of EAS were collapsed to represent agreement, neutral response, and disagreement with the latter serving as reference category. CI confidence interval, *df* degrees of freedom, EAS euthanasia and assisted suicide, *N* sample size, Ref. reference category, *SE* standard error, VASI Value-based Stigma Inventory.

* *p*<.05, *** *p*<.001.

**Table S8**

**Stigma related to low expectations of personal growth predicts EAS.**

| Predictor  (*N* = 1492) | Response category | Predictor estimates | | | Predictor evaluation | | | | Model evaluation | | | | |
| --- | --- | --- | --- | --- | --- | --- | --- | --- | --- | --- | --- | --- | --- |
|  |  | *b* (*SE*) | Relative risk ratio  [95% CI] | | Wald’s χ² (*df*=2) | | *p* | | | Likelihood-ratio χ² (*df*=14) | | Pseudo-*R*² (Mc-Fadden) | |
| VASI subscale Personal growth | Disagree (Ref.) |  |  |  | 6.10 | .047* | | 39.33* | | | .014 | |  |
|  | Neutral | 0.092 (0.046) | 1.096 | [1.001, 1.200] |  |  |  |  |  |  |  |  |  |
|  | Agree | 0.102 (0.053) | 1.107 | [0.997, 1.229] |  |  |  |  |  |  |  |  |  |
| Age (years) | Disagree (Ref.) |  |  |  | 5.33 | .069 | |  |  |  |  |  |  |
|  | Neutral | -0.008 (0.004) | 0.992 | [0.985, 1.000] |  |  |  |  |  |  |  |  |  |
|  | Agree | -0.007 (0.004) | 0.993 | [0.985, 1.001] |  |  |  |  |  |  |  |  |  |
| Gender (0/1: female/male) | Disagree (Ref.) |  |  |  | 13.33 | .001** | |  |  |  |  |  |  |
|  | Neutral | 0.313 (0.129) | 1.367 | [1.062, 1.760] |  |  |  |  |  |  |  |  |  |
|  | Agree | 0.459 (0.140) | 1.583 | [1.204, 2.081] |  |  |  |  |  |  |  |  |  |
| Education (0/1: <10 years/ 10 years) | Disagree (Ref.) |  |  |  | 0.52 | .771 | |  |  |  |  |  |  |
|  | Neutral | 0.521 (0.722) | 1.683 | [0.409, 6.933] |  |  |  |  |  |  |  |  |  |
|  | Agree | 0.123 (0.826) | 1.131 | [0.224, 5.710] |  |  |  |  |  |  |  |  |  |
| Education (0/2: <10 years/ >10 years) | Disagree (Ref.) |  |  |  | 3.90 | .142 | |  |  |  |  |  |  |
|  | Neutral | 0.291 (0.758) | 1.338 | [0.303, 5.907] |  |  |  |  |  |  |  |  |  |
|  | Agree | 1.600 (0.812) | 4.953 | [1.009, 24.303] |  |  |  |  |  |  |  |  |  |
| VASI*Education (0/1: <10 years/ 10 years) | Disagree (Ref.) |  |  |  | 0.52 | .770 | |  |  |  |  |  |  |
|  | Neutral | -0.041 (0.061) | 0.960 | [0.851, 1.082] |  |  |  |  |  |  |  |  |  |
|  | Agree | 0.006 (0.069) | 1.006 | [0.878, 1.152] |  |  |  |  |  |  |  |  |  |
| VASI*Education (0/2: <10 years/ >10 years) | Disagree (Ref.) |  |  |  | 2.61 | .272 | |  |  |  |  |  |  |
|  | Neutral | -0.018 (0.065) | 0.982 | [0.864, 1.117] |  |  |  |  |  |  |  |  |  |
|  | Agree | -0.113 (0.071) | 0.893 | [0.778, 1.025] |  |  |  |  |  |  |  |  |  |

Results of the multinomial logistic regression analyses with the VASI subscale Personal growth, age, gender, educational attainment, and the interaction of VASI*education as predictors of psychiatric EAS. Response categories of EAS were collapsed to represent agreement, neutral response, and disagreement with the latter serving as reference category. CI confidence interval, *df* degrees of freedom, EAS euthanasia and assisted suicide, *N* sample size, Ref. reference category, *SE* standard error, VASI Value-based Stigma Inventory.

* *p*<.05, ** *p*<.01, *** *p*<.001.
